# Please Don't! The Automatic Extrapolation of Dangerous Intentions

**DOI:** 10.1371/journal.pone.0049011

**Published:** 2012-11-14

**Authors:** Alessia Tessari, Giovanni Ottoboni, Andrea Mazzatenta, Arcangelo Merla, Roberto Nicoletti

**Affiliations:** 1 Department of Psychology, University of Bologna, Bologna, Italy; 2 ITAB - Institute for Advanced Biomedical Technology, Foundation University G. d'Annunzio, Chieti, Italy; 3 Department of Neuroscience and Imaging, University of Chieti-Pescara, Chieti, Italy; 4 Department of Communication Sciences, University of Bologna, Bologna, Italy; Baycrest Hospital, Canada

## Abstract

Facial emotions and emotional body postures can easily grab attention in social communication. In the context of faces, gaze has been shown as an important cue for orienting attention, but less is known for other important body parts such as hands. In the present study we investigated whether hands may orient attention due to the emotional features they convey. By implying motion in static photographs of hands, we aimed at furnishing observers with information about the intention to act and at testing if this interacted with the hand automatic coding. In this study, we compared neutral and frontal hands to emotionally threatening hands, rotated along their radial-ulnar axes in a Sidedness task (a Simon-like task based on automatic access to body representation). Results showed a Sidedness effect for both the palm and the back views with either neutral and emotional hands. More important, no difference was found between the two views for neutral hands, but it emerged in the case of the emotional hands: faster reaction times were found for the palm than the back view. The difference was ascribed to palm views' “offensive” pose: a source of threat that might have raised participants' arousal. This hypothesis was also supported by conscious evaluations of the dimensions of valence (pleasant-unpleasant) and arousal. Results are discussed in light of emotional feature coding.

## Introduction

Human beings use to live in social groups and undergo complex patterns of social interactions in daily life. Indeed, human communication requires to spend energies in understanding the meaning of other people's actions and time in watching and interpreting the signals and the actions of others [1]. We can make inferences about other people's state of mind and intentions because of an ability going under the name of “social cognition” [2], [3]. In the contexts of social communication, interaction with the environment and survival-related behaviour, gaze direction is a particularly salient feature in grabbing and orienting attention [4], [5], [6], [7], [8]. Congruently, it has been shown that emotional facial expressions are important social sources of information about others' emotion and mental states [9] too. Eye gaze and emotional expression have been proved to be also important cues for anticipation of biologically and socially important events [10]. In particular, some studies have demonstrated that negative emotional expressions, such as fear and anger, are both rapidly analyzed in the brain at around 120 ms [11], [12] and evoke fast and automatic responses [13], [14]. As to angry facial expressions, Holmes and colleagues [15] have reported that they automatically orient attention in both anxious and non-anxious individuals, when they are compared to neutral, positive or negative facial expressions. The authors suggested that when anger is the source of threat, it may be linked with negative arousal.

De Gelder and colleagues extensively studied another category of biologically salient stimuli: the body postures. They demonstrated that emotional body postures can be automatically processed in both healthy and brain damaged patients either with or without awareness [16]–[18]. Body features such as facial expression, bodily posture and eye gaze rapidly alert observers by attracting and orienting their attention, because they provide individuals with an evolutionary tool for becoming aware of environmentally relevant stimuli concerning others' intentions.

In line with the literature on socially-relevant body features, this study aimed at testing whether a specific body part - eyes and faces apart - might exert the same effect in grabbing attention. In particular, we wanted to assess whether hands in an “intention-to-act” context might differentially affect participants' performance in an attention-related task. So far, it has been demonstrated that not only eyes [5]–[7] but also hands or feet can orient observer's attention [19], [20], [21] independently of any emotional content. It was demonstrated that observers do not code hands automatically on the base of their laterality (i.e. their right or left nature), but, on the base of the position (side) each hand usually affords when represented within a typical body representation (from here originates the name *Sidedness* for this effect). The effect was shown to be dependent of the view of the stimulus: according to the back or palm view, the body representation was represented respectively as facing towards or facing away from the observer. For example, when hands with upright fingers were shown from the palm view they were represented as belonging to a body facing the observer, whereas hands shown from the back view were coded as they belonged to a body facing away from the observer.

So far, the stimuli used to study the Sidedness displayed only static and neutral postures in frontal, canonical view [22]. The use of such stimuli, however, provide limited evidence on the modulatory effect that hands may convey when they embed the information about hand's owner intention to move or to act offensively against the observer. The present study aimed at filling the gap. Indeed, we tested if hands in a threatening posture might be processed differently than the static ones because treated as a negative feature to the same extent as, for example, angry faces [15], [23].

We tried to give the hands a treating value by providing them the aspect of hands that “are-going-to-act”. This manipulation was based on the reports showing that static photographs of human body convey information about implied motion [24]–[25]. Indeed some studies showed that observers are able to extract dynamic information from static stimuli by using the stored internal representation of dynamic information [24]–[27]. In the specific context of hand processing, Urgesi and colleagues [26] reported that static snapshots of a hand suggesting a grasping action activate the same neural systems elicited in both understanding and executing that very same grasping action. Kourtzi and Kanwisher [25] also showed an involvement of medio-temporal/medial superior temporal cortex (i.e. brain areas involved in the visual analysis of motion) during observation of static photographs with implied motion when compared to static photographs.

Therefore, due to the ability to extract movements from implied motions, in the present experiment, we presented frontal hands (as in [19], [20]) and hands rotated along their radial – ulnar axes. We assumed that hands in implied offensive motor attitude toward the observer (i.e. rotated hands), might be differentially processed and affect the Sidedness effect. The frontal hands, instead, being emotionally neutral should not alter the Sidedness effect.

## Experiment

The experimental condition “a” was intended as a control condition in which we presented neutral hand stimuli (i.e. hands in a frontal view) in both palm and back views. In the experimental Condition ”b”, similar hands were rotated along their vertical axes in order to give a sense of intention to act. The hands were rotated in a way that they looked like moving hands. In the case of the back view the hand looked like moving toward something away from the observer (e.g., for reaching an object); whereas in the palm view, the hand looked like moving toward the observer in a sort of “offensive” attitude. In both cases, the movements appeared to be towards the midline of a body which the hands can be represented as belonging to.

In analogy with studies concerning negative emotional expressions, such as fear and anger [13], [14], we hypothesized that, since rotated hands from the palm view might look “offensive” for the observer, (see the threatening value analysis in the method section), participants might respond differently compared to the back views. In particular, we expected a significant difference in the reaction times in response to rotated back view than the palm views (i.e. the supposed threatening stimuli). On the contrary, if the rotated hands do not convey any information about the intention to act and any emotional feature, results should be similar to the ones obtained with “static” and frontal hands.

**Figure 1:**
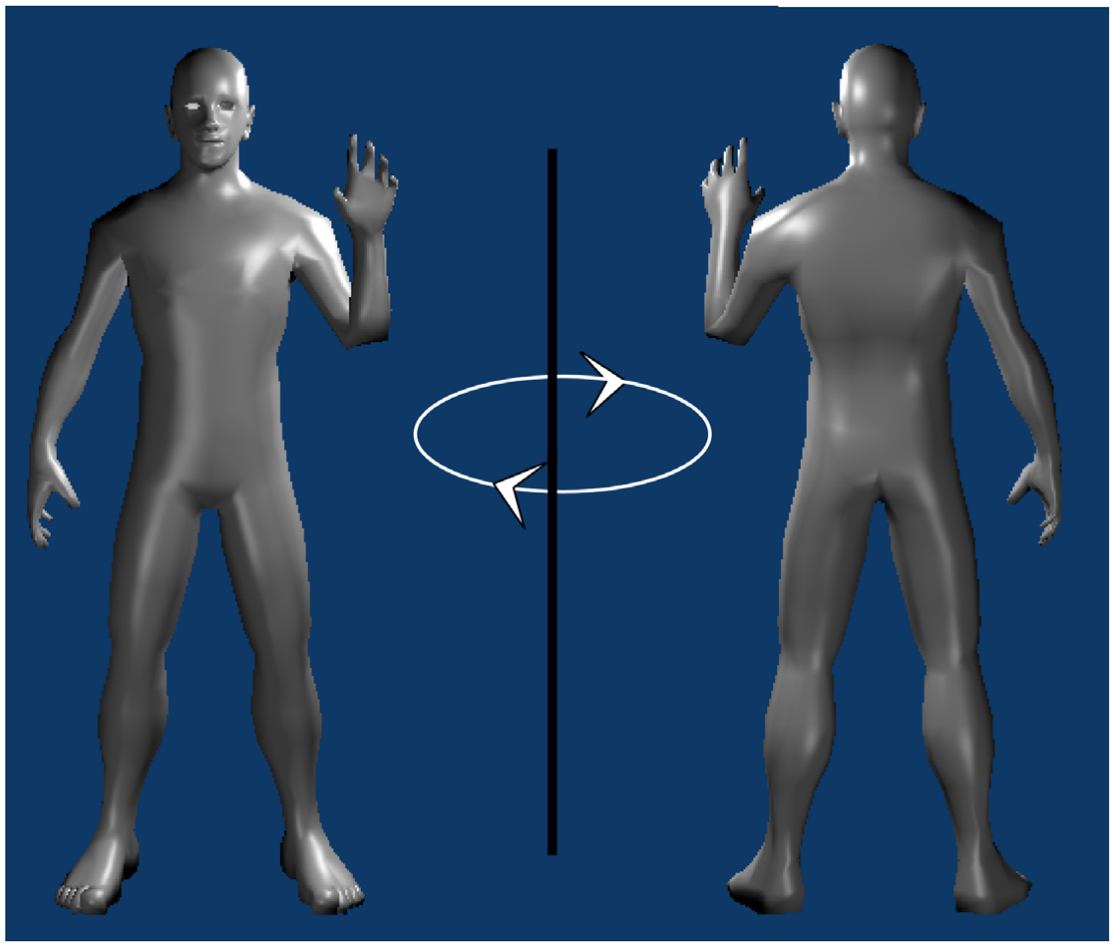
The figure provides a visual representation of the concept of “Sidedness”. A hand always generates a spatial code based on the side it is imagined with respect to the body of reference. The case here reported shows a left hand shifts from a “right sidedness” to a “left sidedness” (we thank Rory O'Sullivan for the Blender UniHuman character model used in this work. UniHuman is available at http://unihuman.yolasite.com/).

To study the different meaning convey by the static and rotated hands, we used a modified Simon paradigm, the Sidedness task [19]. In this task, hands were centrally presented with a coloured (red or blue) circle over the centre of the stimulus. Participants were instructed to press one of the two lateralized keys in response to the colour of the circle. As in the classical Simon task [28], the information about the spatial feature of the stimulus (conveyed in our case by each hand), even though task-irrelevant, gets coded. This way, the spatial feature generates a code which interacts with the response codes by facilitating (when the two codes correspond) or slowing down (when the two codes do not correspond) the responses to the task-relevant features of the stimuli (that is the colour of the superimposed circle). As said in the introduction, when hands are shown, what observers code does not regard hand's laterality, but the spatial position (i.e. the *side*) that, according to its view and posture, each hand would afford within a body representation (i.e. its *Sidedness*). Palm view hands with upright fingers are, for instance, represented as belonging to a body facing the observer. In this configuration, the right hand is represented on the left side of the body and so on the left side of the observer; differently, the left hand would be represented on the right side of the body and, consequentially on the left side of the observer (see Figure 1).

Therefore, for example, when a left response is required in the case of a red circle, reaction times (RTs) are faster when the red circle is superimposed on a right palm hand (non-corresponding condition) than on a left palm hand (corresponding condition). On the contrary, when a back view hand is shown, it is represented as belonging to a body facing away the observer with the left hand laying on the left side (corresponding condition) and the right hand on the right side (non-corresponding condition) of the represented body and of the observer as well. This produces in turn faster RTs when left responses are provided for red circle superimposed on left back hands (corresponding condition) compared to when they are superimposed to right back hands (non-corresponding condition). We expected to observe the Sidedness effect for both the emotionally neutral and the emotionally negative conditions (Conditions a and b, respectively), but also a significant difference between the views (i.e. back and palm) in Condition b where supposed threatening stimuli were shown. It is important to remind that hands are task-irrelevant features in the Sidedness paradigm, and they are automatically and implicitly coded and no explicit judgment is required on them. To be sure to use emotionally relevant stimuli, we investigated their affective values using the Self-Assessment Manikin (SAM) [29], [30] a non-verbal method for assessing reports on affective experiences, that has been shown to covary also with physiological and behavioral emotional reactions (e.g. [31], [32], [33]).

### Methods

#### Participants

Ten students of the University of Bologna were tested in Condition a (mean age = 24, SD = 3.42, 6 male), and 18 new students in the Condition b (mean age = 25, SD = 3.16, 5 male). All of them were right handed, according to the Edinburgh Inventory Test [34], with a normal or corrected-to-normal vision, and naïve to the purpose of the experiment. They were recruited among the students attending the Department of Psychology and took part voluntarily and with no reward to the experiment.

#### Ethics Statement

The experiment was approved by the Psychology Department's ethical committee of the University of Bologna, and subjects provided a written informed consent.

#### Stimuli and Apparatus

**Figure 2:**
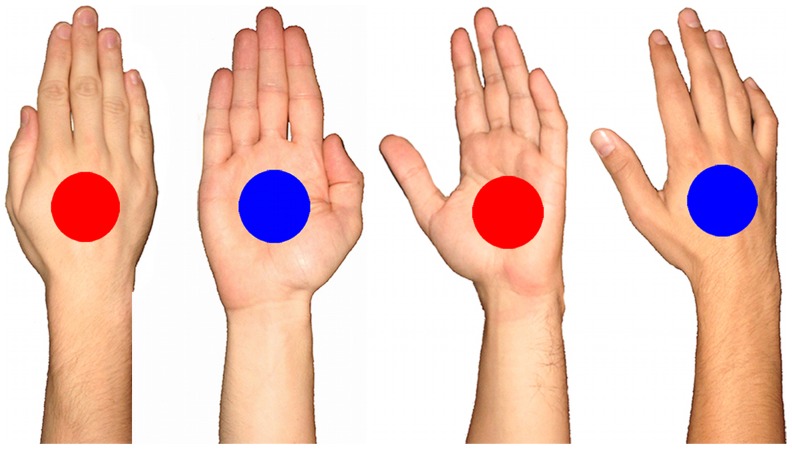
Hand stimuli used in Experiments 1a (on the left) and 1b (on the right): Examples for both the palm and the back views are shown.

The stimuli were photographs of both right and left hands from back and palm views presented in the canonical posture [22] in Condition a and rotated toward the midline along their radial-ulnar axes (slightly 30°) in Condition b (see Figure 2). The stimuli were first scored in an independent rating procedure: 10 raters evaluated the stimuli according to the degree of “implied motion” on a 9-levels scale. Later, the same stimuli were evaluated by 11 different raters (mean age = 22 SD = 1.78, 4 male) for the dimensions of valence (pleasant-unpleasant) and arousal according to the Self-Assessment Manikin (SAM) [29], [30]. SAM figures range from a sad-face figure to a happy-face figure, representing the unpleasant to pleasant dimension, when evaluating valence of the stimuli, and from a calm to an aroused figure when evaluating arousal. Subjects can chose among 5 manikin figures or a value between two manikins resulting in a 9-point rating scale for each dimension. For the instructions we precisely followed those of IAPS [35]. All the ratings were performed in a paper-and-pencil modality and coloured stimuli, of the same size of the ones used in both the conditions a and b, were presented sequentially in a counterbalance order across raters. Orientation (frontal vs. oriented hands) and View (back vs. palm) were the main factors in the analyses.

**Implied motion.** The stimuli of Condition b (i.e. oriented hands, mean = 6.08) transmitted more sense of motion than those of Condition a (i.e. frontal hands, mean = 1.28): Orientation, F(1, 8) = 43.36, MSE = 415.68, p<.001. The other factor (View) and their interaction were not significant (all p>.05).
**Valence.** We only found a significant Orientation×View interaction (F(1,10) = 7.11, MSE = 2.05, p<.05) as oriented palm hands (mean = 4.45) resulted less pleasant than the other stimuli (oriented back hands = 5.1, frontal back hands = 4.8, and frontal palm hands = 5).
**Arousal.** Palm view hands resulted overall more activating (mean = 2.95) than back hands (mean = 2.5) on the arousal dimension (View: F(1,10 = 6.18, MSE = 2.51, p<.05). The other factor and their interaction were not significant (Ps>.05).

In both the conditions hands were presented with forearm and the fingers grouped together (Figure 2). The hands were centrally presented on a 15″ computer screen within 23°×9° of visible angle A red or blue circle was superimposed in the middle of the hand that corresponded to the centre of the screen and to the fixation point. The stimuli were created with Adobe Photoshop (Version 7) software. The experiment was run using a personal computer Pentium III, 512 Mb. The experiment was run with E-Prime 1.1 (SP3) software (Psychology Software Tools Inc.), whereas, the results were analysed by using SPSS software (IBM SPSS Inc.).

### Procedure

Each image lasted on the screen for 100 ms in both Experiments 1a and 1b. Participants were required to respond as quickly as possible according to the colour of the circle by pressing one of two keyboard keys (“X” and “.”) placed, respectively, on the left and the right side of the body midline. Colour-response key associations were counterbalanced across subjects. Each right or left stimulus was presented with either a superimposed red or a blue circle 30 times (for a total amount of 120 trials per view) in each block. Two separated blocks, one for the palm and one for the back view hand stimuli, were presented in a counterbalance order between subjects, resulting in a total amount of 240 experimental trials. Each block was also preceded by a training session of 8 trials. Feedback on RTs, errors and omissions was given at the end of each trial.

The analysed factors were *View* (back vs. palm view) and *Correspondence* (Corresponding pairings vs. Non-corresponding pairings), as the within-subjects factors, and *Condition* (Condition a vs. Condition b), as the between-subjects factor.

### Results

In both Conditions a and b mean correct reaction times (RTs) and error rates (ERs) were the dependent variables. In order to measure the Sidedness effect, responses were coded as corresponding (i.e., the laterality of the response hand corresponded to that of the hand stimulus) and non-corresponding pairings (i.e., the laterality of the response hand did not correspond to that of the hand stimulus). Data filtering was as follows: RTs 2 standard deviations higher or lower than the overall participant's mean (Condition a = 3.1% and Condition b = 3.7%) for corresponding and non-corresponding parings in each block were excluded from the analyses. They only differed in the amount of outliers between corresponding (mean = 1.73) and non-corresponding pairings (mean = 2,41), F(1,26) = 6.46, MSE = 11.91, p<.05. Then RT analyses were only conducted for error-free trials. Data were submitted to a 2×2×2 ANOVA for repeated-measures.

#### RTs

Significant effects of View (F(1,26) = 6.01, MSE = 1637,48 p<.05), Correspondence (F(1,26) = 5.77, MSE = 381.30, p<.05) and their interaction (F(1,26) = 49.78, MSE = 2704.17, p<.001) emerged. The Correspondence×View interaction represents the Sidedness effect: faster RTs for the corresponding pairings than the non-corresponding ones (two-tailed paired-samples t-test t(27) = 6.13, p<.001) are found when the back view is shown; on the contrary, RTs for the corresponding pairings are slower than those for the non-corresponding ones (two-tailed paired-samples t-test t(27) = 3.43, p<.005) when the palm view is shown. The Condition factor was not significant (F = 0.07, p>.05) but the Correspondence×Condition and View×Correspondence×Condition interactions were significant (F(1,26) = 6.968, MSE = 460.327, p<.05 and F(1,26) = 5.605, MSE = 304.489, p<.05, respectively). The Correspondence×Condition interaction was due to the lack of difference between the corresponding (mean = 335 ms) and non-corresponding pairings (mean = 334 ms) in Condition a, and to corresponding pairings (mean = 328 ms) being faster than the non-corresponding ones (mean = 336 ms) in Condition b.

Given the significant triple-interaction we analysed the two conditions separately to better describe the trend of the factors. In Condition a none of the main factors View and Correspondence was significant (*F*(1,9) = 1.28, *MSE* = 724.07, *p*>.05, *F*(1,9) = 0.098, *MSE* = 1.44, *p*>.05, respectively) but their interaction was (*F*(1,9) = 23.03, *MS* = 464.27, *p* = .001). A paired-samples T-test was carried out for the Correspondence factor for the back and palm view separately. The factor was significant for both the back and the palm view (*t*(9) = 4.17, *p* = .005, t(9) = 3.30, *p*<.0025, respectively). The corresponding pairings between the stimulus hand and the response hand were faster than the non-corresponding ones in the back view condition, whereas RTs presented the reversed pattern in the palm view condition (i.e. RTs for the non-corresponding pairings were faster than those for the corresponding ones; See Table 1). This result pattern denotes the Sidedness effect.

**Table 1:** Mean RTs (in ms) and ERs as a function of conditions (hand views) and corresponding and non-corresponding pairings for Condition a and b.

	Condition a		Condition b	
*Pairings*	Back view	Palm view	Back view	Palm view
		*Reaction*	*Times*	
Corresponding	336 (41)	333 (37)	324 (20)	330 (23)
Non-corresponding	342 (40)	328 (35)	346 (30)	325 (20)
		*Errors*		
Corresponding	4.4 (4.5)	5.0 (3.9)	1.9 (2.2)	4.3 (2.6)
Non-corresponding	3.3 (2.7)	3.7 (3.5)	7.3 (4.6)	2.8 (2.0)

Standard deviations are reported in brackets.

The same main factors were also analyzed in Condition b. Each factor was significant (View: *F*(1,17) = 8.94, *MSE* = 999.23, *p*<.005; Correspondence (1,17) = 12.662, *MSE* = 1175,68, *p*<.005) and also their interaction (*F*(1,17) = 46.63, *MSE* = 3376,43, *p*<.001). The Sidedness effect emerged as in Condition a: The corresponding pairings were faster than the non-corresponding ones in the back view condition (one-tailed paired-sample t-test, t(17) = 6.34, p<.001), whereas RTs showed the reversed pattern in the palm view condition (one-tailed paired-sample t-test, t(17) = 2.19, p<.025; See Table 1). It is important to note that the View factor was significant in both the main analysis (data from Conditions a and b collapsed) and it was probably at the origin of the significant triple-interaction View×Correspondence×Condition. This effect emerged for the first time in the literature of the Sidedness effect [19], [20], [21]: as hypothesised RTs to palm views were overall faster than those to back views (mean RTs for the back view = 335 ms, *SD* = 22 ms, mean RTs for the palm view = 328 ms, *SD* = 22 ms).

#### ERs

The Correspondence×Condition, View×Correspondence and View×Correspondence×Condition interactions reached significance (F(1,26) = 13.54, MSE = 63.56, p = .001, F(1,26) = 14.39, MSE = 80.76, p = .001, and F(1,26) = 12.82, MSE = 71.91, p = .001, respectively). More ERs were made for corresponding parings (mean = 5.06%) than non-corresponding (mean = 3.11%) in Condition b (Bonferroni corrected t-test, t(17) = 3.69, p<.005), but no difference was found in Condition a (Bonferroni corrected t-test, t(9) = 1.87, p>.025). Moreover, the View×Correspondence interaction revealed a Sidedness effect: While participants were more accurate in the corresponding compared to non-corresponding pairings (error mean = 3.17% vs. error mean = 5.32%) for the back view (two-tailed t-test, *t*(27) = 3.39, *p*<.005), for the palm view their level of accuracy was higher for non-corresponding than corresponding pairings (error mean = 3.24% vs. error mean = 4.64%; two-tailed *t*(27) = 2.87, *p*<.025). See Table 1.

Due to the significant triple interaction we also performed separated analyses for Conditions a and b with View and Correspondence as main factors. None of the Factors nor their interaction reached significance in Condition a (all Ps>.05). However, Correspondence and View×Correspondence were significant in Condition b (F(1,17) = 13.62, MSE = 68.06, p<.005, and F(1,17) = 32.29, MSE = 213,56, p<.001, respectively). Participants were more accurate with the corresponding (mean = 1.94%) than the non-corresponding pairings (mean = 7.33%) when the back view was displayed (paired-samples t-test, t(17) = 5.49, p<.001), but the reverse pattern emerged for the palm view (mean corresponding pairings = 4.27% vs. mean non-corresponding pairings = 2.78%, paired-samples t-test, t(17) = 2.61, p<.05). This interactions reveals again the presence of the Sidedness effect. The View factor also showed a trend (F(1,17) = 4.03, MSE = 22.22, p = .06) with less error for the palm (mean = 3.53%) than the back view (mean = 4.64). This result pattern was in line with that of the RTs.

### Discussion

RT results indicated a Sidedness effect in both the conditions: The RTs for hands seen from the back are faster when the laterality of the hand stimulus corresponds to the side of the response; conversely, faster RTs are produced when the laterality of the hand stimulus does not correspond to the side of the response for the palm view. Results are in accordance with the notion that automatic, pre-attentive spatial hand coding occurs on the base of Sidedness relation of the hand stimulus with respect to a representation of a body which the hand is connected to (i.e. the side of the hand in relation to a body of reference), and this, in turn, generates a compatibility effect with the side of the response [19], [20], [21]. As regard ERs, the Sidedness effect only emerged in Condition b, for the supposed emotional stimuli (i.e. oriented hands).

Interestingly, in the control condition (i.e., Condition a), when stimuli were supposed to be emotionally neutral no difference emerged between back and palm views. However, in Condition b a *new* result emerged for both RTs and ERs: Participants responded faster and more accurately to palm views compared to back views. As assumed in our hypothesis and demonstrated by the rating, the oriented hands (both palm and back views) conveyed information about implied motion. This quality might have allowed only palm view hands to appear as hands acting toward the observer and thus resulting in a sort of “threatening and dangerous” stimulus, as also suggested by the SAM results for valence [29], [30]: indeed, the oriented palm view hands looked as more negative/unpleasant than the other hand stimuli. We also considered the possibility that the differences in RTs, accuracy and SAM ratings might have been related to the “unusuality” of the stimuli and that the perceptual evaluation might be responsible of the different responses. However, a separate “natural-unnatural” rating ruled out this alternative. Indeed, we performed a rating on the perceptual evaluation of both frontal and oriented hand stimuli. We asked 17 participants to rate each single picture for its truthfulness/“naturality”. Picture were presented sequentially in a counterbalanced order across participants and had to be evaluated on a 5-levels scale (1 = unnatural, 5 = very natural). Orientation (frontal vs. oriented hands) and View (back vs. palm views) were the main factors. Only the main factor Orientation was significant (F(1,16) = 6.95, MSE = 17.50, p<.05) as oriented hands (mean = 3.34) were scored to be more natural than frontal hands (mean = 2.32). The other factor and the interaction were not significant (all Ps>.05).

It is important to note that our results (i.e. faster RT for the negative emotional stimuli) are in line with other studies showing a preferential processing of negative stimuli when attentional visual paradigm are used (e.g. facial expression or eyes; [36]–[41]). These studies showed that threatening facial expressions are processed more efficiently than positive or neutral expressions suggesting that more attentional and processing resources are allocated to negative than positive stimuli. However, this is not in contrast with other research showing exactly the opposite pattern (i.e. slower RTs for negative stimuli; e.g. [42]–[45]) where categorization tasks were carried out. The difference might depend on the paradigms used and the investigated processes. Indeed, in the former cases task-irrelevant emotional stimuli are processed implicitly and affect performance in the main task, and negative emotional stimuli might only increase more rapidly arousal. In contrast, in the latter cases the categorization (i.e. recognition) required detailed, explicit analysis of the emotional meaning of the stimuli and happy expression might be more easy to visually discriminate than negative one [45].

## General Discussion

The ability to detect the intentions and the emotions of the people around us have adaptive implication for anticipating the consequences of their behavior, or, in more extreme cases, for perceiving whether their aggressive action is directed toward us. It is known that “body language” observation can produce emotional responses and induces modification in the observer's behavior (see [46], for a review). However, in order to better study this issue we investigated whether the incidental perception of hand postures might be a socially-relevant information in term of valence and potential actions.

To achieve our aim, we decided to use photographs of frontal and oriented hand postures, by assuming that the oriented hands, due to the information about implied motion they convey, might have looked as hands “potentially” acting. Previous studies [24], [25] have demonstrated that the brain is prone to perceive motion even in static stimuli and that observers are able to extract the dynamic information they convey by using the stored internal representation coming from the dynamic examples [27]. In particular, we were interested in the palm view oriented hands, because, looking as hands acting toward the observer, they might have been considered as a potentially emotional negative (i.e. threatening) stimulus.

In particular, we used frontal and slightly oriented hand posture to test whether they might generate a spatial code able to interact with that of Sidedness (see [19], [20], [21] and introduction for a detailed description). Results indicated that the Sidedness effect emerged for either frontal and oriented hands: Participants were faster to respond to visually presented hands that corresponded to the same side of space, from the observer's point of view, as the participant's responding hand. Results are in line with what was proposed by Ottoboni and colleagues [19], [20], [21] about the automatic completion of a hand with an (imaginary) body, and, with the idea that, regardless of its left or right nature, hand is coded by referring it to a body that faces away or towards the observer. This happens because the presentation of a hand, even if task unrelated, activates the system that defines the local relations between the body parts (the hands in this case) in a perceptual format (i.e. structural description of the body; [47], [48]).

However, a more interesting and new result emerged in Condition b: Faster RTs were found when participants responded to the palm view oriented hands compared to the back view hands. We hypothesised that this result might be due to the arousal increment linked to this peculiar stimulus being perceived as biologically and emotionally negative. The oriented palm hands conveying a potential action toward the observer, might have been evaluated as a potential threatening stimulus. Indeed, the rating we performed on the stimuli, not only showed that oriented hands implied motion more than frontal hands, but also that palm view oriented hands were rated as more unpleasant compared to the other stimuli in SAM [29], [30]. However, besides SAM judgements being conscious and overtly required and Sidedness task only investigating covert, pre-attentive processing of hands, it is well established that SAM dimension of pleasure and arousal covaries with behavioural measures [31]–[33].

Behavioural data seem to suggests that oriented hands, seen from the palm view, might look like hands in a “threatening” pose, i.e. as if they were going to hit the observer, and they can be supposed to represent “biologically primitive expressions” of emotions that induce an increment in arousal [49], [50], [51], [52], [53], [54]. As already found for other body features (e.g. [6], [7], [8], [9], [14]) these emotionally stimuli have a special effect on grabbing and orienting attention compared to the neutral ones. From an evolutionary perspective it is not surprising that these hands, as they look like in an offensive pose, might induce higher arousal levels and orient attention more quickly, and these data appear in line with what already demonstrated in the domain of emotional face coding [13]–[15]. In our case, we can suppose that the oriented palm hands might result in a strong adaptive emotional signal in primary communication systems, which elicits a response for activating consequently rapid defense actions or escape reactions. Arousing negative stimuli, pre-attentively detected, might have elicited a rapid response action and this reaction has an evolutionary value as to its survival implications. Indeed, it has been suggested that pre-attentive mechanisms allow for rapid focusing of attention and cognitive resources on emotionally relevant environmental situations ([55], [56] for a review).

Therefore, hands, faces and whole bodies appear to be multi-dimensional stimuli conveying many important social and motivational signals. In the domain of motion, our results can be also interpreted in light of theory of mind and the understanding of intentions (See [57], for a review). It has been suggested that the brain, plausibly adapted along evolution, is able to detect biological motion in order to extract intentions and to predict the future actions of other individuals [58]. Participants might have covertly detected the implied biologically-relevant motion in the stimuli and extracted the intention of a possible action in another individual. Indeed, it is known that the same representations, governing action control and action production, are also involved in the perception of actions performed by others [59], [60]. However, intentions always imply both goal representations (ends) and body movements (means). According to Prinz's distinction, in this experiment we were in the domain of action perception, that proceeds from movements to goals, and it is used to recover the others' intentions from the perceived body-part movements (see [61] for a review). What is interesting in this study is the fact that emotional intention might have been automatically extrapolated from an “implied” movement. In this way, the motor system appears to be able to connect perception of simple body-parts to intentional actions. By eliciting action oriented reactions, on the bases of the mechanism of shared motor representations, the motor system might allow us to understood and even predicted other people actions [62]–[65]. It is not a surprise that hands in isolation elicited such emotional responses as it is assumed that what is automatically recalled from the hand stimuli is an entire body representation [19], [20], [21]. If this were true, indeed, our results might be in line with the literature on emotional bodily postures, because they showed an automatic processing even when attention is deployed elsewhere or perception is totally unaware [18], [19]. Interesting results on perception of bodily emotions come also from recent neuroimaging data. It has been shown that perceiving emotional bodily expressions [66]–[71] and even angry hands [71] elicits activations in both the regions underlying motor representations (e.g. the premotor cortex, superior temporal sulcus, supramarginal gyrus) and the regions involved in emotional processing (e.g. insula) [66]–[71]. Despite this, it is still under debate whether the critical role in emotional action understanding is played by a resonance in the motor program necessary to execute an action (e.g. the mirror neuron system; see [72] for a review) or by the interaction between the emotion-processing areas and the action-related network, that is a resonance in the emotional system responsible for the affective modulation of the motor program.

Future studies might better investigate either the physiological and the brain components related to the presentation of these stimuli.

## References

[1] Barresi J, Moore C (1996) Intentional relations and social understanding. Behav Brain Sci 19: 107–154.

[2] Baron-Cohen S (1995) Mindblindness: An essay of autism and theory-of-mind. Cambridge, MA: MIT Press.

[3] Kleinke CL (1986) Gaze and eye contact: A research review. Psychol Bull 100: 78–100. 3526377

[4] Emery NJ (2000) The eyes have it: The neuroethology, function and evolution of social gaze. Neurosci Biobehav Rev 24: 581–604. 10940436 10.1016/s0149-7634(00)00025-7

[5] Lagton SRH, Bruce V (2000) You must see the point: Automatic processing of the cues to the direction of social attention. J Exp Psychol: Human Per Perf 27: 747–57. 10.1037//0096-1523.26.2.747 10811173

[6] Driver J, Davis G, Ricciardelli P, Kidd P, Maxwell E, et al (1999) Gaze perception triggers reflexive visuospatial orienting. Vis Cog 6: 509–540.

[7] Friesen CK, Kingstone A (1998) The eyes have it! Reflexive orienting is triggered by nonpredictive gaze. Psychon Bull & Rev 4: 490–495.

[8] Nummenmaa L, Calder AJ (2009) Neural mechanisms of social attention. Trends Cog Sci 13: 135–143. 10.1016/j.tics.2008.12.006 19223221

[9] Baron-Cohen S, Riviere A, Fukushima M, French D, Hadwin J, et al (1996) Reading the Mind in the Face: A Cross-cultural and Developmental Study. Vis Cog 3: 39–59.

[10] Ekman P, Oster H (1979) Facial expression of emotion. Ann Rev Psychol 30: 527–554.

[11] Eimer M, Holmes A (2002) An ERP study of the time course of emotional face processing. Neuroreport 13: 427–431. 11930154 10.1097/00001756-200203250-00013

[12] Kawasaki H, Kaufman O, Damasio H, Damasio A, Granner M, et al (2001) Single-neuron responses to emotional visual stimuli recorded in human ventral prefrontal cortex. Nat Neurosci 4: 15–16. 11135639 10.1038/82850

[13] Armony JL, Dolan RJ (2002) Modulation of spatial attention by fear-conditioned stimuli: An event-related fMRI study. Neuropsychologia 40: 817–826. 11900732 10.1016/s0028-3932(01)00178-6

[14] Holmes A, Villeumier P, Heimer M (2003) The processing of emotional facial expression is gated by spatial attention: Evidence form event-related brain potential. Cog Brain Res 16: 174–184. 10.1016/s0926-6410(02)00268-9 12668225

[15] Holmes A, Richards A, Green S (2006) Anxiety and sensitivity to eye gaze in emotional faces. Brain Cog 20: 282–294. 10.1016/j.bandc.2005.05.002 16510226

[16] de Gelder B (2006) Toward the neurobiology of emotional body language. Nat Rev Neurosci 7: 242–249. 16495945 10.1038/nrn1872

[17] de Gelder B, Hadjikhani N (2006) Non-conscious recognition of emotional language. Neuroreport 17: 583–586. 16603916 10.1097/00001756-200604240-00006

[18] Tamietto M, Geminiani G, Genero G, de Gelder B (2007) Seeing fearful body language overcomes attentional deficits in patients with neglect. J Cog Neurosci 19: 445–454. 10.1162/jocn.2007.19.3.445 17335393

[19] Ottoboni G, Tessari A, Cubelli R, Umiltà C (2005) Is handedness recognition automatic? A study using a Simon-like paradigm. J Ep Psychol: Human Per Perf 31: 778–89. 10.1037/0096-1523.31.4.643b 16131249

[20] Tessari A, Ottoboni G, Symes E, Cubelli R (2010) Hand processing depends on the implicit access to a spatially and bio-mechanically organized structural description of the body. Neuropsychologia 48: 681–688. 19958784 10.1016/j.neuropsychologia.2009.11.020

[21] Tessari A, Ottoboni G, Baroni G, Symes E, Nicoletti R (2012) Is access to the body structural description sensitive to a body part's significance for action and cognition? A study of the sidedness effect using feet. Exp Brain Res 218: 1–11. 22402752 10.1007/s00221-012-3045-4

[22] Cooper LA, Shepard RN (1975) Mental transformations in the identification of left and right hands. J Exp Psychol: Human Per Perf 104: 48–56. 1141835

[23] Adams RBJ, Kleck RE (2003) Perceived gaze direction and the processing of facial displays of emotion. Psychol Sci 14: 644–647. 14629700 10.1046/j.0956-7976.2003.psci_1479.x

[24] Freyd JJ (1983) The mental representation of movement when static stimuli are viewed. Percep Psychophys 33: 575–581. 10.3758/bf03202940 6622194

[25] Kourtzi Z, Kanwisher N (2000) Activation in human MT/MST by static images with implied motion. J Cog Neurosci 12: 48–55. 10.1162/08989290051137594 10769305

[26] Urgesi C, Moro V, Candidi M, Aglioti SM (2006) Mapping implied body actions in the human motor system. J Neurosci 26: 7942–7949. 16870739 10.1523/JNEUROSCI.1289-06.2006 PMC6674209

[27] Blakemore SJ, Decety J (2001) From the perception of action to the understanding of intention. Nat Rev Neurosci 2: 561–567. 11483999 10.1038/35086023

[28] Simon JR (1969) Reaction towards the source of stimulation. J Exp Psychol 81: 174–176. 5812172 10.1037/h0027448

[29] Lang PJ (1980) Behavioral treatment and bio-behavioral assessment: computer applications. In: Sidowski JB, Johnson JH, Williams TA, editors. Technology in Mental Health Care Delivery Systems. Ablex Publishing, Norwood, NJ. pp. 119–137.

[30] Lang PJ, Bradley MM (1994) Measuring emotion: The self-assessment manikin and the semantic differential. J Behav Ther Exp Psychiatry 25: 49–59. 7962581 10.1016/0005-7916(94)90063-9

[31] Lang PJ, Greeenwald MK, Bradley MM, Hamm AO (1993) Looking at pictures: Affective, facial, visceral, and behavioral reactions. Psychophysiol 30: 261–273. 10.1111/j.1469-8986.1993.tb03352.x 8497555

[32] Lang PJ, Bradley MM, Cuthbert BN (1990) Emotion, attention, and the startle reflex. Psycholog Rev 97: 377–395. 2200076

[33] Bradley MM, Greenwald MK, Petry MC, Lang PJ (1992) Remembering pictures: Pleasure and arousal in memory. J Exp Psychol: Lear Mem Cog 18: 379–390. 10.1037//0278-7393.18.2.379 1532823

[34] Oldfield RC (1971) The assessment and analysis of handedness: The Edinburgh inventory. Neuropsychologia 9: 97–113. 5146491 10.1016/0028-3932(71)90067-4

[35] Lang PJ, Bradley MM, Cuthbert BN (1997) International Affective Picture System (IAPS). Instruction manual and affective ratings. Technical Report A-4, The Center for Research in Psychophysiology, University of Florida.

[36] Baumeister RF, Bratslavsky E, Finkenauer C, Vohs KD (2001) Bad is stronger than good. RevGen Psychol 5: 323–370.

[37] Fox E, Lester V, Russo R, Bowles RJ, Pichler A, et al (2000) Facial expressions of emotions: Are angry faces detected more efficiently? Cog Emo 14: 61–92. 10.1080/026999300378996 PMC1839771 17401453

[38] Eastwood JD, Smilek D, Merikle PM (2001) Differential attentional guidance by unattended faces expressing positive and negative emotion. Perc Psychophysics 63: 1004–1013. 10.3758/bf03194519 11578045

[39] Hansen CH, Hansen RD (1988) Finding the face in the crowd: An anger superiority effect. J Pers Soc Psychol 54: 917–924. 3397866 10.1037//0022-3514.54.6.917

[40] Fox E, Griggs L, Mouchlianitis E (2006) The detection of fear-relevant stimuli: Are guns noticed as quickly as snakes? Emotion 7: 691–696. 10.1037/1528-3542.7.4.691 PMC2757724 18039035

[41] Fox E, Damjanovic L (2006) The eyes are sufficient to produce a threat superiority effect. Emotion 6: 534–539. 16938095 10.1037/1528-3542.6.3.534 PMC1852642

[42] Tamietto M, Latini Corazzini L, de Gelder B, Geminiani G (2006) Functional asymmetry and interhemispheric cooperation in the perception of emotions from facial expressions. Exp Brain Res 171: 389–404. 16374630 10.1007/s00221-005-0279-4

[43] Billings LS, Harrison DW, Alden JD (1993) Age differences among women in the functional symmetry for bias in facial affect perception. B Psychonomic Soc 31: 317–320.

[44] Leppänen JM, Tenhunen M, Hietanen JK (2003) Faster choice-reaction times to positive than to negative facial expressions: The role of cognitive and motor processes. J Psychoph 17: 113–123.

[45] Ducci L (1981) Reaction times in the recognition of facial expressions of emotion. Italian J Psych 8: 183–193.

[46] Buxbaum LJ, Coslet HB (2001) Specialized structural description for human body parts: Evidence from autotopagnosia. Cog Neuropsychol 18: 289–306. 10.1080/02643290126172 20945217

[47] Grezes J, de Gelder B (2008) Social perception: understanding other people's intentions and emotions through their actions. In Striano T, Reid V, editors. Social Cognition: Development, Neuroscience and Autism. Oxford: Wiley-Blackwell. pp. 67–78.

[48] Sirigu A, Grafman J, Bressler K, Sunderland T (1991) Multiple representations contribute to body knowledge processing. Evidence from a case of autotopagnosia. Brain 114: 629–642. 2004260 10.1093/brain/114.1.629

[49] Glacher J, Adolphs R (2003) Processing of the arousal of subliminal and supraliminal emotional stimuli by the human amygdala. J Neurosci 23: 10274–10282. 14614086 10.1523/JNEUROSCI.23-32-10274.2003 PMC6741000

[50] Rippon G (1990) Individual differences in electrodermal and electroencephalographic asymmetries. Int J Psychophysiol 8: 309–320. 2338410 10.1016/0167-8760(90)90021-5

[51] Sequeira H, Roy JC (1993) Cortical and hypothalamo-limbic control of electrodermal responses. In Roy JC, Boucsein W, Fowles DC, editors. Progress in Electroderal Research. New York: Plenum. pp. 93–114.

[52] Dawson ME, Schell AM, Filion DL (2000) The electrodermal system. In Cacioppo JT, Tassinary LG, Berntson GG editors. Handbook of psychophysiology. New York: Cambridge University Press. pp. 200–223.

[53] Costa RM, Esteves F (2008) Skin conductance responses to visual sexual stimuli. Int J Psychophysiol 67: 64–69. 18006098 10.1016/j.ijpsycho.2007.10.005

[54] Snowdon CT, Ziegler TE, Schultz-Darken NJ, Ferris CF (2006) Social odours, sexual arousal and pairbonding in primates. Philos Trans R Soc Lond B 361: 2079–2089. 17118925 10.1098/rstb.2006.1932 PMC1764847

[55] Robinson MD (1998) Running from William James' bear: a review of preattentive mechanisms and their contribution to emotion experience. Cogn Emot 12: 667–696.

[56] Tamietto M, de Gelder B (2010) Neural bases of the non-conscious perception of emotional signals. Nat Rev Neurosci 11: 697–709. 20811475 10.1038/nrn2889

[57] Blakemore SJ, Sarfati Y, Bazin N, Decety J (2003) The detection of intentional contingencies in simple animations in patients with delusions of persecution. Psychol Med 33: 1433–1441. 14672252 10.1017/s0033291703008341

[58] Gobbini MI, Koralek AC, Bryan RE, Montgomery KJ, Haxby JV (2007) Two takes on the social brain: a comparison of theory of mind tasks. J Cog Neurosci 19: 1803–1814. 10.1162/jocn.2007.19.11.1803 17958483

[59] Prinz W (1997) Perception and action planning. Eur J Cogn Psychol 4: 1–20.

[60] Prinz W (2002) Experimental approaches to imitation. In Meltzoff AN, Prinz W, editors. The imitative mind: Development, evolution, and brain bases. Cambridge: Cambridge University Press. pp. 143–162.

[61] Prinz W (2006) Representational foundation of intentional actions. In Knoblich G, Thornton IM, Grosjean M, Shiffran M, editors. Human Body perception from the inside out. New York: Oxford University Press. pp. 393–411.

[62] Csibra G (2007) Action mirroring and action understanding: An alternative account. In Haggard P, Rosetti I, Kawato M, editors. Sensorimotor Foundations of Higher Cognition. Attention and Performance XXII. Oxford: Oxford University Press. pp. 435–459.

[63] Flanagan JR, Johansson RS (2003) Action plans used in action observation. Nature 424: 769–771. 12917683 10.1038/nature01861

[64] Kilner JM, Vargas C, Duval S, Blakemore SJ, Sirigu A (2004) Motor activation prior to observation of a predicted movement. Nat Neurosci 7: 1299–1301. 15558063 10.1038/nn1355

[65] Knoblich G, Prinz W (2001) Recognition of self-generated actions from kinematic displays of drawing. J Exp Psychol: Human Per Perf 27: 456–465. 10.1037//0096-1523.27.2.456 11318059

[66] de Gelder B, Tamietto M (2007) Affective blindsight. Scholarpedia 2: 3555.

[67] Carr L, Iacoboni M, Dubeau MC, Mazziotta JC, Lenzi GL (2003) Neural mechanisms of empathy in humans: A relay from neural systems for imitation to limbic areas. Proc Natl Acad Sci U S A 100: 5497–5502. 12682281 10.1073/pnas.0935845100 PMC154373

[68] Decety J, Chaminade T (2003) Neural correlates of feeling sympathy. Neuropsychologia 41: 127–138. 12459211 10.1016/s0028-3932(02)00143-4

[69] de Gelder B, Snyder J, Greve D, Gerard G, Hadjikhani N (2004) Fear fosters flight: A mechanism for fear contagion when perceiving emotion expressed by a whole body. Proc Natl Acad Sci U S A 101: 16701–16706. 15546983 10.1073/pnas.0407042101 PMC528902

[70] Grèzes J, Pichon S, de Gelder B (2007) Perceiving fear in dynamic body expressions. NeuroImage 35: 959–967. 17270466 10.1016/j.neuroimage.2006.11.030

[71] Grosbras MH, Paus T (2006) Brain networks involved in viewing angry hands or faces. Cerebral Cortex 16: 1087–1096. 16221928 10.1093/cercor/bhj050

[72] Rizzolatti G, Craighero L (2004) The mirror-neuron system. Annu Rev Neurosci 27: 169–192. 15217330 10.1146/annurev.neuro.27.070203.144230

